# Nursing students’ perceptions and attitudes towards dementia care in Namibia

**DOI:** 10.4102/hsag.v29i0.2692

**Published:** 2024-08-20

**Authors:** Nestor Tomas, Andrias M. Mangundu

**Affiliations:** 1 Department of General Nursing Science, Faculty of Health Science and Veterinary Medicine, University of Namibia, Rundu, Namibia

**Keywords:** aged, dementia, Namibia, nursing students, patient care, perception, witchcraft

## Abstract

**Background:**

Dementia is a significant public health concern and a leading cause of disability and dependency among elderly individuals globally. However, there is currently limited research examining the perceptions and attitudes of healthcare providers, including nursing students, in Namibia.

**Aim:**

This study aimed to examine nursing students’ perceptions and attitudes of caring for patients with dementia.

**Setting:**

The study was conducted at two university campuses in Namibia.

**Methods:**

A quantitative correlational design was employed to conveniently recruit 196 third- and fourth-year nursing students between April and August 2023, utilising the Geriatric In-Hospital Nursing Care Questionnaire (GerINCQ) online survey. Data were analysed using SPSSv28 for descriptive and inferential statistics.

**Results:**

The perception score ranged from 2.26 to 2.43, indicating negative attitudes and perceptions towards dementia care. The highest mean perception of 2.43 (95% CI = 2.3–2.55) was on reacting to challenging behaviour and the lowest was on professional responsibility 2.26 (95% CI = 2.12–2.4). Correlations were observed between age and performed interventions (*r* = 0.66; *p* = 0.01) and gender and dementia-sensitive care (*r* = 0.52; *p* = 0.01).

**Conclusion:**

The study revealed unfavourable attitudes and perceptions towards dementia care among nursing students, contrary to the optimistic anticipation. The results highlight the urgent need to develop and integrate dementia care strategies and practices into nursing education curricula.

**Contribution:**

This study provides valuable data for informing quality assurance initiatives aimed at improving nursing training in dementia care.

## Introduction

Dementia is a rapidly growing public health issue and a significant contributor to disability and dependence in the elderly population globally (Lisko et al. 2019; Scott, Kugelman & Tulloch 2019; WHO 2015). As an umbrella term, dementia refers to a group of conditions characterised by a gradual decline in cognitive functions (Nilsson, Annersten Gershater & Bengtsson 2022; Scott et al. 2019). Research has demonstrated that nurses who have received education in dementia care tend to display favourable attitudes and perspectives when it comes to providing care for individuals with dementia. However, there is limited research that has been conducted on how nursing students perceive dementia care in most African countries (Musoke et al. 2021; Naidoo, Waggie & Van Wyk 2020).

Research indicates that by 2050, approximately 22% of the global population will be aged 60 years and above (Di Lorito et al. 2020). Moreover, it is projected that around 80% of elderly individuals worldwide will be from low- and middle-income countries (Muhsin et al. 2020). These statistics highlight a projected significant rise in chronic illnesses and cognitive impairment conditions such as dementia. Currently, there are over 57 million people living with dementia (Dawson & Comas-Herrera 2022). Of those diagnosed with dementia and Alzheimer’s disease, about 2.13 million individuals are found in sub-Saharan Africa (Lynch 2020). The existing literature reveals that despite the global increase in dementia burden, the African continent bears over 24% of the total dementia burden while having access to merely 3% of qualified health professionals (Bonnechère & Sahakian, 2020).

Dementia not only causes cognitive decline, such as memory loss and deterioration in functions but also impairs an individual’s ability to plan and organise social activities (Nilsson et al. 2022).

Because of patients refusing or struggling to accept nursing care (Keuning-plantinga et al. 2022), as well as the progressive decline in physical and cognitive functions, the presence of behavioural and psychological symptoms adds complexity and burden to dementia care (Christianson et al. 2021). The available evidence indicates that individuals diagnosed with dementia are more likely to experience deterioration in both physical and cognitive capabilities, heightened susceptibility to infections, incontinence issues and prolonged hospital admissions (Keuning-plantinga et al. 2022; Kwak et al. 2022; Scerri, Innes & Scerri 2019). Similarly to nurses, nursing students play a crucial role in managing challenging behaviours, such as agitation, aggression, resistance to care, and confusion, in patients with dementia who often have comorbidities (Evripidou et al. 2019). Therefore, nursing students, as part of the healthcare team, take care of people with dementia during their clinical placements. However, they may not receive adequate preparation during their undergraduate programmes. Furthermore, the lack of support during clinical placements may negatively affect nursing students’ perceptions of caring for patients with dementia. Strom, Engedal and Andreassen (2021) clearly state that there is insufficient emphasis on the diagnosis and management of dementia in the training of healthcare professionals including nurses. Therefore, it is important to understand the perspectives of healthcare professionals regarding the care of patients with dementia. Studies have shown that nurses with training in dementia care (Laura et al. 2022) and those in areas with improved dementia infrastructure exhibit positive attitudes and perceptions towards caring for patients with dementia.

Africa is facing significant challenges related to dementia care. Several studies reported that healthcare providers in sub-Saharan Africa have negative perceptions of patients with dementia, often attributing the condition to witchcraft (Brooke & Ojo 2019; Jacobs et al. 2022). Additional research has suggested that dementia is often perceived as a condition that primarily affects individuals of white ethnicity (Roche et al. 2020).

As a member of the Southern Africa Development Community (SADC), Namibia is ranked 79th in the world in terms of total deaths caused by dementia, accounting for about 176 deaths, 1.04% (World Health Rankings 2024). According to Pretorius (2019), Namibia has only one dementia care facility, Alzheimer’s Dementia Namibia (ADN), located in the coastal town of Swakopmund, providing specialised services for individuals with dementia. The facility has a limited number of admission beds.

Literature indicates a dearth of research on the perception of dementia care among nursing students in Africa (Musoke et al. 2021; Naidoo et al. 2020). Considering the absence of any studies conducted on this subject in Namibia, this study aims to explore the perceptions of nursing students regarding the care provided to individuals with dementia at two selected satellite campuses of the esteemed University of Namibia.

## Research methods and design

### Design and setting

This study employed a quantitative correlational design. The research was carried out among nursing students at two health campuses affiliated with a renowned university in Namibia. These campuses are located in prominent towns within the Kavango and Khomas regions: two of the three regions in Namibia with intermediate hospitals. These hospitals cater to patients from approximately nine regions. The campuses offer a 4-year honours degree programme in nursing science and enrol around 700 nursing students. These students are assigned to different public health facilities in these regions to provide dementia care and other services to patients admitted for acute conditions.

### Sample and recruitment

Data were collected between April and August 2023. The researcher contacted respondents through academic WhatsApp groups, providing them with a link to access the questionnaire online. While 190 responses were collected through an online survey, the researcher personally approached respondents without smartphones at the study location and obtained data from six individuals using a printed version of the questionnaire. The questionnaire included information about the study’s purpose and significance, along with a request for consent to participate. Respondents were informed that they could withdraw from the study at any time. To indicate consent, respondents who chose to take part in the study clicked the ‘Agree’ button on the online questionnaire. Data were collected from 196 third- and fourth-year nursing students recruited through a convenient sampling technique to readily available respondents who met the inclusion criteria. The sample size of 188 students was determined using Solvin’s formula: *n* = *N*/(1+*N*e2) (Tomas, Munangatire & Nampila 2023), with a confidence level of α = 0.05. However, considering the utilisation of online survey, a total of 196 respondents were recruited to ensure sufficient statistical power (Andrade 2020). The eligibility criteria were willing senior nursing students from the third and fourth years, who had received training in geriatric nursing, from two health university campuses in Namibia. These students were selected because of their advanced stage of study and proximity to completion. Furthermore, these students had more frequent opportunities to provide direct care for patients with dementia in medical and surgical departments.

### Measure

In order to evaluate the nursing care provided to dementia patients by nursing students, the study employed the validated Dutch Geriatric In-Hospital Nursing Care Questionnaire (GerINCQ) (Keuning-Plantinga et al. 2022). The GerINCQ (Keuning-plantinga et al. 2022) had three sections with 67 items, and a Cronbach’s alpha score of α = 0.70. Demographic data were measured with five questions (age, sex, marital status, religion and year of study).

#### Attitudes

To measure attitudes, nursing students were asked to evaluate their sentiments concerning various aspects of nursing care, encompassing their experience and degree of engagement. Nursing students’ attitudes towards dementia care were measured using attitude subscale using 14 items (α = 0.84). A question such as ‘Do you communicate in plain language with patients who have dementia?’ was utilised to assess attitudes.

#### Perceptions of care

Participants were requested to indicate their level of agreement with the provided statements regarding the interventions they carried out for the care of their patients with dementia. The perceptions of care among nursing students were assessed by evaluating the interventions performed. These interventions focussed on areas such as preventing malnutrition and physical movement restrictions (α = 0.78); ageing-sensitive care was evaluated with a 13-item scale (α = 0.86). For example, respondents were asked to rate their level of satisfaction regarding the respectful treatment of patients with dementia. Additionally, a 16-item scale (α = 0.71) was utilised to gauge respondents’ attitudes towards caring for elderly patients, e.g. perceived difficulty in managing restless patients with dementia. The study also assessed participants’ sense of professional responsibility through a 12-item scale (α = 0.88), which included questions such as the extent to which they feel accountable for addressing behavioural issues in dementia patients. Furthermore, a series of 20 items (α = 0.79) were used to examine nursing students’ perceptions on handling patients exhibiting challenging behaviours, e.g. ‘I use physical force to calm the situation’.

The tool used a Likert scale with 4 points to rate both attitudes and perception subscales. The rating ranged from 1 (never) to 4 (always). The questionnaire was presented in English and it took about 20 min – 40 min to complete.

To assess the construct validity, a rigorous exploratory factor analysis was conducted on the 68 items specifically designed to measure the knowledge of registered nurses. These items were carefully subjected to principal component analysis with varimax rotation to ensure accurate evaluation. Two key assessments, namely the Kaiser–Meyer–Olkin (KMO) and Bartlett’s test of sphericity, were utilised to evaluate the validity of the tool. The KMO value yielded a commendable result of 0.74, while the *p*-value for Bartlett’s test of sphericity was found to be statistically significant (*p* = 0.00), as detailed by Shrestha (2021). The total score spanned from a minimum of 67 points to a maximum of 272 points, with the highest score indicating a positive perception and the lowest score indicating a negative perception. It is noteworthy that aggregate mean scores of 3 reflected a positive perception and attitudes regarding the care of dementia patients, whereas scores ranging from 1 to 2.9 were indicative of negative perceptions and attitudes.

### Data analysis

The Statistical Package for the Social Sciences (SPSS) v28 was utilised for data analysis. The analysis process commenced with the computation of descriptive statistics, which provided an overview of the background characteristics of the respondents. The data were then summarised into means and percentages. Inferential statistics were employed to examine the relationship between variables. Specifically, Spearman’s rho was employed to determine whether there were any significant (*p* < 0.05) correlations between the domains and background variables.

### Ethical consideration

The study received approval from the Ethical Committee of the School of Nursing and Public Health (ref no: SoN 11/2023). In addition, the Ministry of Health and Social Services (ref no: 22/3/1/2) ensured that ethical considerations were met. The researchers adhered to core ethical principles, including respect for individuals, beneficence and justice, all of which are underpinned by the imperative to protect human rights throughout the research process (Brink, Van der Walt & Van Rensburg 2018). Before proceeding to the research questions, respondents were prompted to provide written consent by signing the informed consent form or by selecting the ‘agreement button’ in the provided hyperlink for the online survey. Respondents had the convenience of completing the survey in their own homes, thereby guaranteeing their privacy. Participation was entirely voluntary, and no personally identifiable information was required, ensuring complete confidentiality and anonymity. The electronic data collected were exclusively accessible to the researchers. This study upheld the principles of the revised Declaration of Helsinki.

## Results

### Demographic characteristics

A total of 196 respondents were included in the study with a response rate of 100% (Table 1). The median age category was 23–28 years, which accounted for nearly 50% (*n* = 92) of the respondents. Majority of the respondents (68.90%, *n* = 135) were female, indicating a predominance of female nursing students in training. Furthermore, more than half of the respondents (54.10%, *n* = 106) were in their fourth year of study, with the satellite campus having a higher number of respondents (60.2%, *n* = 118) compared to the main campus (39.8%, *n* = 78). The majority of respondents (88.30%, *n* = 173) were identified as Christians, followed by Jehovah’s Witnesses with 9.2% (*n* = 18) and other religions with 2.6% (*n* = 5).

**TABLE 1:** Respondents’ demographic data (*N* = 196).

Variables	Frequencies (*n*)	%
**Age (years)**		
18–20	15	7.7
21–22	65	33.2
23–28	92	46.9
29–34	18	9.2
≥ 35	6	3.1
Median age: 23–28	-	-
**Gender**		
Male	61	31.1
Female	135	68.9
**Religion**		
Christian	173	88.3
Jehovah Witness	18	9.2
Others	5	2.6
**Year level**		
Third year	90	45.9
Fourth year	106	54.1
**Campus of study**		
Satellite campus	118	60.2
Main campus	78	39.8

### Overall students’ perceptions and attitudes

**FIGURE 1:**
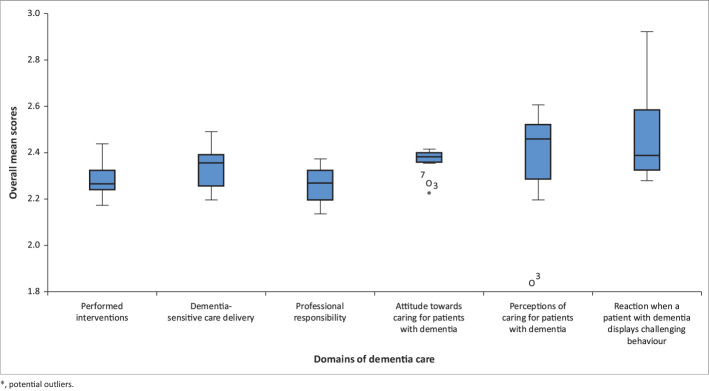
Box-and-whisker plot.

As shown in Table 2, the item means vary from 2.26 to 2.43 and the weighted means from 2.12 to 2.24. The data are summarised in a box-and-whisker plot, as presented in Figure 1. The domain reaction when a patient displays challenging behaviour (2.43 [95% CI = 2.3–2.55]) has the most extensive spread and the domain professional responsibility with the lowest score (2.26 [95% CI = 2.12–2.4]).

**TABLE 2:** An overview of psychometric properties of the Geriatric In-Hospital Nursing Care Questionnaire per domain.

Domains	Item mean	95 % Confidence interval		Cronbach’s alpha	95 % Confidence interval	
		Lower	Upper		Lower	Upper
Performed interventions	2.28	2.16	2.40	0.39	0.25	0.51
Dementia-sensitive care delivery	2.32	2.20	2.45	0.49	0.38	0.59
Professional responsibility	2.26	2.12	2.40	0.60	0.51	0.67
Attitude towards caring for patients with dementia	2.36	2.24	2.48	0.51	0.41	0.61
Perceptions of caring for patients with dementia	2.39	2.24	2.53	0.09	−0.10	0.27
Reaction to patients with dementia challenging behaviour	2.43	2.30	2.55	0.52	0.41	0.61

As shown in Table 2, the mean score of the domain performed interventions is 2.28 (95% CI = 2.16–2.4). Based on the results, the authors are 95% confident that the mean perceptions of caring for patients with dementia were 2.39 (95% CI = 2.24–2.53), 2.36 (95% CI =2.24–2.48) for attitude towards caring for patients with dementia and 2.32 (95% CI =2.2–2.45) for providing dementia-sensitive care.

### Correlation between demographic characteristics and study domains

At scale level, a positive Spearman’s rank correlation coefficient was found between the domain performed interventions and some of the demographic variables (Table 3). A moderate positive correlation existed between age and performed interventions (*r* = 0.66; *p* = 0.01) and professional responsibility (*r* = 0.59; *p* = 0.03), gender and dementia-sensitive care (*r* = 0.52; *p* = 0.01), religion and performed intervention (*r* = 0.50; *p* = 0.00), and year level and attitudes on care of dementia patient (*r* = 0.53; *p* = 0.01). These relationships suggest that age may play a significant role in the types of interventions performed and the level of professional responsibility demonstrated. Furthermore, gender seems to influence the approach to dementia-sensitive care, while religion appears to impact the engagement in performed interventions. Lastly, year level appears to correlate with attitudes towards the care of dementia patients.

**TABLE 3:** Correlation between demographic characteristics and study domains.

Variables	Age	Gender	Religion	Marital status	Year level	Residence	Campus of study
**Performed interventions**							
Rho	0.66	0.45	0.50	0.53	0.36	0.50	0.55
*p*	0.01	0.00	0.00	0.04	0.00	0.00	0.07
**Dementia-sensitive care delivery**							
Rho	0.47	0.52	0.41	0.44	0.48	0.55	0.46
*p*	0.01	0.01	0.06	0.00	0.02	0.00	0.02
**Professional responsibility**							
Rho	0.59	0.59	0.49	0.53	0.65	0.56	0.52
*p*	0.03	0.00	0.03	0.00	0.00	0.00	0.02
**Attitude towards caring for patients with dementia**							
Rho	0.46	0.33	0.40	0.49	0.53	0.48	0.77
*p*	0.00	0.00	0.07	0.02	0.01	0.04	0.01
**Perceptions of caring for patients with dementia**							
Rho	0.45	0.38	0.43	0.36	0.43	0.62	0.42
*p*	0.02	0.01	0.03	0.01	0.05	0.01	0.00
**Reaction to patients with challenging behaviour**							
Rho	0.48	0.50	0.52	0.48	0.51	0.55	0.44
*p*	0.00	0.03	0.00	0.02	0.00	0.00	0.03

Note: *p* < 0.05.

The weakest correlation was reported between gender and performed interventions (*r* = 0.456; *p* = 0.002), as well as between gender and reaction to challenging behaviour (*r* = 0.501; *p* = 0.033).

## Discussion of results

To mitigate situations in which nurses may refrain from caring for patients with dementia, particularly when they display challenging behaviour (Keuning-plantinga et al. 2022), evaluating nursing students is recognised as a crucial factor in fostering a positive perception and attitude towards providing care for patients with dementia. The study’s findings indicate that female nursing students had a more positive attitude and perception towards dementia sensitivity care compared to their male counterparts. This implies that being a female nursing student has a positive impact on the delivery of nursing care to patients with dementia. Many studies viewed caring as a female characteristic (Graham 2022; Van der Cingel & Brouwer 2021). The notion that caring is a female responsibility has a deep root in many African cultures, as women traditionally took on the role of caregivers (Harling et al. 2020; Yakubu, Fuseini & Holroyd 2022). This suggests that within African societies, women are generally expected to be the primary caregivers for older adults, children and spouses, as opposed to their male counterparts. These findings emphasise the gendered cultural and societal expectations surrounding women’s traditional caregiving roles and imply that women have an innate talent for caregiving. Equally important is the association of gender and professional responsibility highlighted in this research. This underscores the significance of providing dementia-sensitive care. By implications, the current findings support that female nursing students are associated with respecting the rights of individuals with disabilities as outlined in the United Nations Convention (Kuliga, Berwig & Roes 2021). This emphasis on professional accountability serves as a crucial factor in enhancing the quality of care and support for those living with dementia, ultimately contributing to a more inclusive and compassionate society.

Consistent with prior research (Aljezawi et al. 2022), the present study revealed that a significant proportion of participants consisted of younger students within the 23–28 years age bracket. Conversely, other studies (Harling et al. 2022; Yakubu et al. 2022) found a higher proportion of respondents in the 40–49 years age category. The variation could be attributed to factors such as differences in the curriculum. For example, dementia is taught in the third and fourth years at one university, whereas another university teaches it in the first and second years (Aljezawi et al. 2022). The duration of programmes may also contribute to these differences, as institutions offering a 2-year dementia curriculum may have dissimilar outcomes compared to those with a 4-year curriculum.

The Spearman’s rank correlation coefficient indicated a positive correlation between the performed intervention and age. Similar findings have been reported in other studies, where age was found to influence attitudes towards individuals with dementia (Musoke et al. 2021; Wang, Xiao & Huang 2020). In the context of this study, both performed interventions and age are believed anecdotally to contribute to a more positive attitude among nursing students towards caring for elderly individuals with dementia. Within many cultures, there is a longstanding tradition of young adults showing respect and care towards their senior family members. Iyare, Imafidon and Abudu (2022) found that younger individuals tend to exhibit greater levels of obedience and respect towards individuals living with dementia when compared to adults.

Demographic data, such as age, have been identified and reported as a significant predictor, accounting for a substantial 36.7% change in professional behaviour and commitment (Duran et al. 2021). The study found a moderate association between the age of the respondents and their level of professional responsibility in the context of making appropriate decisions related to dementia care. The findings revealed that age was identified as a crucial factor in ensuring that professionals could effectively navigate complex ethical dilemmas and conflicts within the nursing profession. In alignment with the existing literature, the findings underscore the significance of nursing professional values in guiding nurses to make difficult decisions and resolve conflicts while upholding the ethical values of the profession (Chen et al. 2021).

Furthermore, research has found that nursing students who are religious may perceive older adults as their grandparents and, as a result, treat them with respect and care (Aljezawi et al. 2022; Attafuah et al. 2022; Harling et al. 2020; Yakubu et al. 2022). Honouring older adults is highly regarded, as providing care to the elderly is seen as serving God in the Christian faith (Yakubu et al. 2022). This consensus may explain the significant relationship established in this study between religion and the interventions performed, given the Christian affiliation of the respondents.

This study supports findings from prior research (Aljezawi et al. 2022; Evripidou et al. 2019), indicating a moderate positive correlation between the academic year of nursing students and their attitudes towards providing care for dementia patients. This suggests that the level of education of students is essential in shaping positive attitudes towards individuals with dementia. Therefore, in this study, it is believed that senior students with greater clinical exposure, theoretical knowledge and training may be attributed to positive attitudes. However, no significant differences were observed between training and academic levels. This suggests that dementia training across different education levels does not yield any significant differences in students’ perceptions and attitudes. However, the findings should be interpreted with caution, as the presence of a connection between variables is not enough to definitively claim that one variable caused the other, even if the relationship was significant.

This study’s findings coincide with Keuning-Plantinga et al. (2020), who found that nursing students often perform general preventive interventions, such as nursing interventions to prevent falls, delirium and malnutrition in patients with dementia. Nonetheless, Feast et al. (2020) disagree that nursing students rarely engage in interventions associated with dementia care, such as providing activities for patients with dementia, making it difficult to effectively mitigate complications.

Surprisingly, the overall nursing students in this study displayed negative behaviours and perceptions towards patients with dementia. This result is consistent with Keuning-Plantinga et al. (2020), which found a mean score of 2.61–3.46 corresponding to the current study. In the recent study, respondents had negative perceptions dealing with challenging dementia behaviour in patients. This could be attributed to a lack of specialised skills among nursing students. It is crucial for nurses to possess the necessary skills and knowledge to understand patients’ unmet needs and effectively address them. In order to provide individualised specialised dementia care, nurses must undergo specialised training (Lundin & Godskesen, 2021). Therefore, it is important for nurses to recognise that challenging behaviours may arise from environmental factors, unmet needs or healthcare providers’ approaches (Christianson et al. 2021; Lodha & Sousa 2018).

Clearly, respondents demonstrated negative perceptions and attitudes towards caring for patients with dementia across all the five domains measured in this study. Interestingly, respondents’ heavy reliance on restraining methods contradicted patients’ rights to freedom and had the potential to cause harm.

### Strengths and limitations

Although this study provided useful data to inform educators and policymakers regarding the need to develop and implement context-based teaching strategies to improve dementia care, it has several limitations. The study employed quantitative methods, using questionnaires that contained only close-ended questions. As a result, students were not given the opportunity to provide extensive explanations. Owing to the employment of convenience sampling in participant recruitment, the results of this study lack generalisability to alternative contexts. Additionally, the analysis was limited to associations, and no regression analysis was performed to confidently determine which factors are most important, which factors can be ignored, and how these factors influence each other. Enhancing the generalisability of the findings can be achieved by utilising a larger multistage sample. Moreover, this study relied on self-report perceptions, making it difficult to understand how patients with dementia experienced nursing care provided by nursing students in Namibia. Future research should be conducted to investigate the various factors that impact and hinder dementia care among nursing students in Namibia.

### Implication on the nursing practice

Contrary to expectations, the research demonstrated that nursing students have negative attitudes and perceptions towards dementia care. These findings carry significant implications for the advancement of curricula and future investigations designed to improve nursing students’ comprehension of dementia care. This is crucial in light of the rising elderly population and the escalating demand for top-tier care for individuals affected by dementia. The study results have significant implications for training strategies and practices within nursing education programmes. The study therefore recommends that faculty should consider developing and incorporating context-based teaching strategies on dementia care and increasing the number of dedicated teaching hours focussed on dementia.

## Conclusion

The study revealed that nursing students had negative perceptions and attitudes towards caring for patients living with dementia. Female nursing students exhibited a more positive attitude and perception towards dementia sensitivity care in comparison to their male counterparts. These results warrant the attention of nurse educators and policymakers to develop a comprehensive support system and training strategy for nursing students, aiming to enhance their proficiency in providing care for patients with dementia. Future research should be conducted to investigate the various factors that impact and hinder dementia care among nursing students.

## References

[CIT0001] Andrade, C., 2020, ‘Sample size and its importance in research’, *Indian Journal of Psychological Medicine* 42(1), 102–103. 10.4103/IJPSYM.IJPSYM_504_19 31997873 PMC6970301

[CIT0002] Aljezawi, M.E., Al Qadire, M., Suliman, M., Al Omari, O. & Khalaf, A., 2022, ‘Undergraduate nursing students’ knowledge of and attitudes toward people with Alzheimer’s disease’, *BMC Geriatrics* 22(1), 691. 10.1186/s12877-022-03389-6 35996080 PMC9394018

[CIT0003] Attafuah, P.Y.A., Amertil, N., Sarfo, J.O., Deegbe, D.A., Nyonator, D., Amponsah-Boama, C. et al., 2022, ‘I decided to attend to him because it’s my duty’: Student nurses perception and attitude towards care of older adults’, *BMC Medical Education* 22(1), 23. 10.1186/s12909-021-03090-z 34998393 PMC8742453

[CIT0004] Bonnechère, B. & Sahakian, B.J., 2020, ‘Can mobile technology help prevent the burden of dementia in low-and mid-income countries?’, *Frontiers in Public Health* 8, 554938. 10.3389/fpubh.2020.554938 33282809 PMC7689265

[CIT0005] Brink, H., Van der Walt, C. & Van Rensburg, G., 2018, *Fundamentals of research methodology for healthcare professionals*, 4th edn., Juta, Cape town.

[CIT0006] Brooke, J. & Ojo, O., 2020, ‘Contemporary views on dementia as witchcraft in sub-Saharan Africa: A systematic literature review’, *Journal of Clinical Nursing* 29(1–2), 20–30. 10.1111/jocn.15066 31531993

[CIT0007] Chen, Q., Su, X., Liu, S., Miao, K. & Fang, H., 2021, ‘The relationship between moral sensitivity and professional values and ethical decision-making in nursing students’, *Nurse Education Today* 105, 105056. 10.1016/j.nedt.2021.105056 34265538

[CIT0008] Christianson, T.M., Hoot, T.J., McLelland, V. & Morris, K., 2021, ‘“All behaviour has meaning”: A qualitative exploration of dementia training of healthcare assistant students’, *Journal of Nursing Education and Practice* 11(2), 28–36. 10.5430/jnep.v11n2p28

[CIT0009] Dawson, W.D. & Comas-Herrera, A., 2022, ‘International dementia policies and legacies of the corona-virus disease 2019 pandemic’, *Public Policy & Aging Report* 32(2), 72–76. 10.1093/ppar/prac008 35996432 PMC9383947

[CIT0010] Di Lorito, C., Bosco, A., Booth, V., Goldberg, S., Harwood, R.H. & Van der Wardt, V., 2020, ‘Adherence to exercise interventions in older people with mild cognitive impairment and dementia: A systematic review and meta-analysis’, *Preventive Medicine Reports* 19, 101139. 10.1016/j.pmedr.2020.101139 32793408 PMC7414005

[CIT0011] Duran, S., Celik, I., Ertugrul, B., Ok, S. & Albayrak, S., 2021, ‘Factors affecting nurses’ professional commitment during the COVID-19 pandemic: A cross-sectional study’, *Journal of Nursing Management* 29(7), 1906–1915. 10.1111/jonm.13327 33794061 PMC8250040

[CIT0012] Evripidou, M., Charalambous, A., Middleton, N. & Papastavrou, E., 2019, Nurses’ knowledge and attitudes about dementia care: Systematic literature review’, *Perspectives in Psychiatric Care* 55(1), 48–60. 10.1111/ppc.12291 29766513

[CIT0013] Feast, A.R., White, N., Candy, B., Kupeli, N. & Sampson, E.L., 2020, ‘The effectiveness interventions to improve the care and Management of people with dementia in general hospitals: A systemic review’, *International Journal of Geriatric Psychiatry* 35(5), 463–488. 10.1002/gps.5280 32011033

[CIT0014] Graham, H., 2022, ‘Caring: A labour of love’, in J. Finch & D. Groves (eds.), *A labour of love: Women, work and caring*, pp. 13–30, Routledge & Kegan Paul, London.

[CIT0015] Harling, G., Payne, C.F., Davies, J.I., Gomez-Olive, F.X., Kahn, K., Manderson, L. et al., 2020, ‘Impairment in activities of daily living, care receipt, and unmet needs in a middle-aged and older rural South African population: Findings from the HAALSI study’, *Journal of Aging and Health* 32(5–6), 296–307. 10.1177/0898264318821220 30600746 PMC6675676

[CIT0016] Iyare, A.E., Imafidon, E. & Abudu, K.U., 2022, ‘Ageing, ageism, cultural representations of the elderly and the duty to care in African traditions’, in J.O. Chimakonam, E. Etieyibo & I. Odimegwu (eds.), *Essays on contemporary issues in African philosophy*, pp. 281–299, Springer, Cham.

[CIT0017] Jacobs, R., Schneider, M., Farina, N., Du Toit, P. & Evans-Lacko, S., 2022, ‘Stigma and its implications for dementia in South Africa: A multi-stakeholder exploratory study’, *Ageing & Society* 44(4), 1–31. 10.1017/S0144686X2200040X

[CIT0018] Keuning-Plantinga, A., Roodbol, P.F., Krijnen, W.P. & Finnema, E.J., 2022, ‘Nurses’ perceptions in caring for people with dementia in Dutch acute hospitals’, *Journal of Clinical Nursing* 31(13–14), 1800–1816. 10.1111/jocn.15458 32780901 PMC9292336

[CIT0019] Kuliga, S., Berwig, M. & Roes, M., 2021, ‘Wayfinding in people with Alzheimer’s disease: Perspective taking and architectural cognition – A vision paper on future dementia care research opportunities’, *Sustainability* 13(3), 1084. 10.3390/su13031084

[CIT0020] Kwak, C., Seo, Y.J., Park, K.H. & Han, W., 2022, ‘Analysis of the knowledge, attitudes, and practice model of healthcare professionals on hearing loss at elderly dementia residences in Korea’, *Healthcare* 10(5), 792. 10.3390/healthcare10050792 35627929 PMC9140935

[CIT0021] Laura, P.A., Dolores, L.F.M., Pedro, G.F.F. & Luis, P.H.P., 2022, ‘Undergraduate nursing students’ knowledge of Alzheimer’s disease and related dementias care’, *Journal of Professional Nursing* 39, 101–108. 10.1016/j.profnurs.2022.01.005 35272816

[CIT0022] Lisko, I., Kulmala, J. ,Annetorp, M., Ngandu, T., Mangialasche, F. & Kivipelto, M., 2019, ‘How can dementia and disability be prevented in older adults: Where are we today and where are we going? can dementia and disability be prevented in older adults’, *Journal of Internal Medicine* 289(6), 807–830. 10.1111/joim.13227 PMC8248434 33314384

[CIT0023] Lodha, P. & De Sousa, A., 2018, ‘Geriatric mental health: The challenges for India’, *Journal of Geriatric Mental Health* 5(1), 16. 10.4103/jgmh.jgmh_34_17

[CIT0024] Lundin, E. & Godskesen, T.E., 2021, ‘End-of-life care for people with advanced dementia and pain: a qualitative study in Swedish nursing homes’, *BMC nursing*, 20, 1–11. 10.1186/s12912-021-00566-7 33743691 PMC7981921

[CIT0025] Lynch, C., 2020, ‘World Alzheimer report 2019: Attitudes to dementia, a global survey: Public health: Engaging people in ADRD research’, *Alzheimer’s & Dementia* 16(S10), e038255. 10.1002/alz.038255

[CIT0026] Muhsin, A.F., Munyogwa, J.M., Kibusi, S.M. & Seif, S.A., 2020, ‘Poor level of knowledge on elderly care despite positive attitude among nursing students in Zanzibar Island: Findings from a cross-sectional study’, *BMC Nursing* 19(1), 1–8. 10.1186/s12912-020-00488-w 33061842 PMC7547516

[CIT0027] Musoke, P., Olum, R., Kembabazi, S., Nantaayi, B., Bongomin, F. & Kaddumukasa, M., 2021, ‘Assessment of the knowledge and attitude towards dementia among undergraduate University Students in Uganda’, *Advances in Medical and Practice Journal* 12, 635–646. 10.2147/AMEP.S301445 PMC8214337 34163279

[CIT0028] Naidoo, K., Waggie, F. & Van Wyk, M.J., 2020, ‘A review of geriatric care training. Africa in the undergraduate nursing and medical curricula at the University of KwaZulu-Natal, South’, *African Journal of Health Professions Education* 12(3), 130–133. 10.7196/AJHPE.2020.v12i3.1349

[CIT0029] Nilsson, M.L., Annersten Gershater, M. & Bengtsson, M., 2022, ‘Registered nurses’ experiences of caring for persons with dementia expressing their sexuality’, *Nursing Open* 9(3), 1723–1730. 10.1002/nop2.1197 35170245 PMC8994936

[CIT0030] Roche, M., Higgs, P., Aworinde, J. & Cooper, C., 2021, ‘A review of qualitative research of perception and experiences of dementia among adults from Black, African, and Caribbean background: What and whom are we researching?’, *The Gerontologist* 61(5), e195–e208. 10.1093/geront/gnaa004 32077938 PMC8276611

[CIT0031] Pretorius, E., 2019, *Living with Alzheimer’s disease in Namibia: The adult child, the older parent and the decision to institutionalise*, Doctoral dissertation, University of Pretoria, viewed n.d., from http://hdl.handle.net/2263/72670.

[CIT0032] Scerri, A., Innes, A. & Scerri, C., 2019, ‘Using appreciative inquiry to implement person-centred dementia care in hospital wards’, *Dementia* 18(1), 190–209. 10.1177/1471301216663953 27758956

[CIT0033] Scott, T.L., Kugelman, M. & Tulloch, K., 2019, ‘How medical professional students view older people with dementia: Implications for education and practice’, *PLoS One* 14(11), e0225329. 10.1371/journal.pone.0225329 31747449 PMC6867636

[CIT0034] Shrestha, N., 2021, ‘Factor analysis as a tool for survey analysis’, *American Journal of Applied Mathematics and Statistics* 9(1), 4–11. 10.12691/ajams-9-1-2

[CIT0035] Strom, B.S., Engedal, K. & Andreassen, L., 2021, ‘Nursing staffs knowledge and attitudes towards dementia’, *Dementia and Geriatric Cognitive Disorder Extra* 9(3), 352–361. 10.1159/000502770 PMC6792425 31616459

[CIT0036] Tomas, N., Munangatire, T. & Nampila, S., 2023, ‘Undergraduate students’ knowledge, attitudes and willingness to receive COVID-19 vaccines: A survey of convenience sample in Namibia’, *SAGE Open Nursing* 9, 23779608231177565. 10.1177/23779608231177565 37250766 PMC10214085

[CIT0037] Van der Cingel, M. & Brouwer, J., 2021, ‘What makes a nurse today? A debate on the nursing professional identity and its need for change’, *Nursing Philosophy* 22(2), e12343. 10.1111/nup.12343 33450124

[CIT0038] Wang, Y., Xiao, L.D. & Huang, R., 2020, ‘A comparative study of dementia knowledge, attitudes and care approach among Chinese nursing and medical students’, *BMC Medical Education* 20, 436. 10.1186/s12909-020-02365-1 33198736 PMC7670709

[CIT0039] World Health Organization, 2015, *World report on ageing and health*, World Health Organization, Geneva, Switzerland.

[CIT0040] World Health Rankings, live longer live better, 2024, *Namibia: Alzheimers & Dementia*, viewed 20 February 2024, from https://www.worldlifeexpectancy.com/namibiaalzheimers-dementia

[CIT0041] Yakubu, Y.H., Fuseini, A.G. & Holroyd, E., 2022, ‘Nurses’ attitudes towards hospitalized older adults in a tertiary care setting in Ghana’, *Nursing Open* 9(4), 2054–2062. 10.1002/nop2.1216 35527338 PMC9190693

